# Right Ventricular Myocardial Strain and Proteomic Analysis of Fetuses With Left Ventricular Hypoplasia: A Cross‐Sectional Study

**DOI:** 10.1002/hsr2.71455

**Published:** 2025-11-19

**Authors:** Jing Ma, Sushan Xiao, Liu Hong, Juanjuan Liu, Shi Jiawei, Yi Zhang, Li Cui, Haiyan Cao, Mingxing Xie, Li Zhang

**Affiliations:** ^1^ Department of Ultrasound Union Hospital, Tongji Medical College, Huazhong University of Science and Technology Wuhan China; ^2^ Clinical Research Center for Medical Imaging in Hubei Province Wuhan China; ^3^ Hubei Province Key Laboratory of Molecular Imaging Wuhan China

## Abstract

**Background and Aims:**

Decreased right ventricular (RV) function in fetuses with left ventricular hypoplasia (LVH) increases the risk of adverse outcome. This study aimed to quantitatively evaluate RV myocardial function prenatally in LVH, and explore the relevant mechanism from protein expression.

**Methods:**

81 singleton fetuses diagnosed with LVH and 81 normal controls were retrospectively included. We used RV global longitudinal strain (RVGLS) derived from two‐dimensional speckle tracking imaging to evaluate RV myocardial function and “XGboost” algorithm to select effective factors affecting RV myocardial function in fetuses with LVH. Bioinformatics analysis was performed for differentially expressed proteins between specimens with LVH and normal fetuses.

**Results:**

In LVH fetuses, RVGLS was significantly lower in fetuses with LVH than in controls (*p* < 0.001). “XGboost” model showed that RV/LV ratio, LV sphericity index, combined with double outlet right ventricle, and LV end‐diastolic dimension z‐score had the greatest effect on RVGLS, with SHAP analysis revealing nonlinear relationships between these factors and RVGLS. Proteomics in LVH fetuses demonstrated 144 differentially expressed RV proteins (68 upregulated, 76 downregulated), enriched in extracellular matrix, cytoskeleton, and DNA replication pathways (GO/KEGG). Notably, dysregulated processes included actin depolymerization, cell cycle control, and MCM complex function, suggesting adaptive RV remodeling at molecular levels. Interobserver reproducibility for strain measurements was excellent (ICC > 0.9).

**Conclusions:**

Impaired RV myocardial function and RV protein spectrum differentiated from normal fetuses have been observed in fetuses with LVH, which shed light on the novel perspective that myogenic developmental abnormalities are not limited to the left heart but also extend to RV cardiomyocytes during the embryonic stage.

## Summary

- Impaired RV myocardial function has been observed in fetuses with LVH.
- Changes in RV tissue protein expression, which refers to cardiomyocyte contraction, potentially provide significant advice in prenatal counseling and management of fetuses with LVH.

## 1 Introduction

Left ventricular hypoplasia (LVH) is characterized by underdevelopment or inadequate growth of the left heart, and encompasses a wide range of conditions, extending from typical hypoplastic left heart syndrome (HLHS) to borderline left ventricle (BLV) [1]. Despite various postnatal surgical approaches, morbidity and mortality remain high as reported in patients with LVH [2, 3, 4]. Reduced right ventricular (RV) function could increase the risk of postnatal death or the need for cardiac transplantation [5]. Generally, fetuses with LVH less often manifest as poor RV performance. However, inadequate systemic perfusion at birth triggers an increase in cardiac output as a compensatory mechanism that can immediately deteriorate RV function. One possible reason is the physiological transition from prenatal to postnatal, this cannot fully explain why the cardiac function has not improved significantly after postnatally intervention, suggesting that other factors, such as cellular remodeling or altered myocardial microstructure, may also be contributing to the persistence of dysfunction.

However, recent studies have not yielded consistent conclusions regarding RV function in fetuses with LVH. Fetal echocardiography is the first‐line imaging modality for diagnosing LVH and assessing cardiac function [6]. In recent years, two‐dimensional‐speckle tracking imaging (2D‐STI) has been demonstrated to be feasible for the evaluation of fetal CHDs in several studies [7, 8, 9, 10].

An association between abnormal myocardial protein expression and inadequate cardiac performance has been proved [11, 12]. Emerging studies have identified potential mechanisms underlying the onset of LVH, including genetic and hemodynamic causes [13, 14, 15]. However, these studies have may primarily investigate the mechanisms of left heart lesions. The RV myocardial pathogensis remains unclear.

Thus, we aimed to: 1) evaluate RV myocardial function in fetuses with LVH using 2D‐STI, 2) compare the proteomics profile of RV myocardium to investigate the mechanism underlying myocardial function changes.

## 2 Methods

### 2.1 Study Population

A total of 81 fetuses diagnosed with LVH who were scheduled for prenatal echocardiography were retrospectively enrolled in this study at Wuhan Union Hospital between January 2016 and July 2022. The LVH group was selected by the criteria of (1) LV end‐diastole dimension (LVEDD) Z‐score < –2.0, or LV end‐diastole length (LVEDL) Z‐score < –2.0; (2) mitral stenosis or atresia; (3) aortic stenosis or atresia. Subject to meeting the first criterion, at least one of the latter two criteria must also be satisfied. The fetuses with double outlet right ventricle (DORV), and right‐dominant unbalanced atrioventricular septal defect(R‐UAVSD) were also included. The control group consisted of fetuses with normal cardiac structure and function, as determined by pre‐ and postnatal echocardiograms. Exclusion criteria in LVH group included:(1) pregnancy complications that may affect cardiac function (diabetes, hypertension, hyperthyroidism/hypothyroidism)；(2) acute infection during pregnancy; (3) twin or multiple pregnancies. Control fetuses (*n* = 81) had normal cardiac morphology and function on prenatal echocardiographic examination. Exclusion criteria in control group included: (1) had fetal chromosomal abnormality, (2) extracardiac anomaly, (3) utero‐placental circulatory insufficiency or intrauterine growth restriction, (4) maternal diseases such as diabetes, hypertension, hyperthyroidism/hypothyroidism, (5) acute infection during pregnancy, (6) twin or multiple pregnancies. Demographic and clinical data were recorded.

For myocardial proteomics studies, three fetal specimens from the LVH group were donated to the center after induction of labor. Three fetal specimens with normal cardiac structure due to inevitable miscarriage or stillbirth in the same period were selected as the control group. Informed consent was obtained from all the families who donated fetal specimens. In the informed consent, families were informed that the specimens may be used for subsequent scientific research, and they all agreed. The ethics committee of Tongji Medical College of Huazhong University of Science and Technology (from 2015 to 2019) and Union Hospital (from 2020 to 2022) approved this study's use of fetal specimens from (S118,0510).

### 2.2 Conventional Fetal Echocardiographic Measurements

Detailed fetal echocardiogram was performed using GE Voluson E10 system (GE Healthcare, USA) with transabdominal probes RM6C (4‐8 MHz) or C2‐9‐D (2–9 MHz) by two experienced fetal echocardiography specialists (H Cao, L Hong), according to the guideline recommended by the International Society of Ultrasound in Obstetrics and Gynecology (ISUOG) [16]. The first fetal echocardiogram was selected for analysis for all enrolled participants. Fetal cardiac structural assessment was performed using segmental analysis. Left and right ventricular end‐diastolic diameter (LVEDD, RVEDD), left and right ventricular end‐diastolic length (LVEDL, RVEDL), foramen ovale (FO) diameter were measured in four‐chamber view (4CV). Global sphericity index (SI) was defined as the ratio of ventricular length to transverse diameters. Diameters of aortic and pulmonary valve annulus (AV, PV), ascending aorta (AAo), main pulmonary artery (MPA), ductus arteriosus (DA) were measured in corresponding standard views. 2D measurements were transformed to the z‐scores adjusting for gestational age [17, 18]. Pulse Doppler was used to obtain peak systolic velocities of aortic valve, pulmonary valve and DA.

### 2.3 Two‐Dimensional Speckle Tracking Imaging (2D‐STI)

2D clips of apical 4CV were captured and stored in Digital Imaging and Communications in Medicine (DICOM) format files. Offline deformation analysis was conducted by two‐dimensional speckle tracking software (Fetal version of TomTec Imaging Systems 2.4, Munich, Germany). A single cardiac cycle was selected via anatomical M‐mode. Three tracing points were sequentially placed on the septal annulus, lateral annulus and apex, after which the endocardial border was automatically outlined frame‐by‐frame throughout the cardiac cycle. Endocardial tracing was manually adjusted when tracking was inadequate. The software automatically provided averaged longitudinal strain‐time curves, from which peak global longitudinal strain (GLS) and six segmental strains (basal, middle, apical segments of the RV free wall and septum), could be derived. Intra‐observer variability was evaluated by having one observer remeasuring after 2 weeks. Interobserver variability was assessed by a second observer who was blinded to the first observer's measurementsbased on the same M‐mode cardiac cycle obtained from the same clip.

### 2.4 Myocardial Proteomic Analysis of FetusesWith Right Ventricular Hypoplasia

RV tissues from LVH and control fetuses were cut for the subsequent proteomics analysis. Identified proteins were categorized by using the software program IPA (Ingenuity Databases, www.ingenuity.com) and UniProt database (http://www.uniport.org/). Differentially expressed proteins were identified by fold‐change filtering (absolute fold change > 1.5) and standard Student's *t* test (*p*‐value < 0.05). Gene ontology (GO) function and Kyoto Encyclopedia of Genes and Genomes (KEGG) enrichment for differentially expressed proteins were used to identify the significantly enriched biological terms and pathways. The KEGG pathway and GO bioinformatics analyses were performed on the Dr. Tom network platform of BGI (http://report.bgi.com) and Sangerbox (http://www.sangerbox.com/tool) [19]. Protein‐protein interaction (PPI) network was used by STRING (string‐db. org) to clarify the interaction network of these differentially expressed proteins and regulators. Cytoscape (http://www.cytoscape.org) (version 3.9.1) [20] was used to visualize this PPI network. To further elucidate the biological processes and pathways in these differential proteins, we used CytoScape with ClueGo plugins to examine Gene Ontology terms and KEGG and Reactome pathways to build a network of the significantly enriched processes.

### 2.5 Statistical Analysis

All data analyses were performed using SPSS version 23 (IBM; Armonk, New York, USA), GraphPad Prism 9.0.0(121) and R, version 4.1.3 (R Core Team (2022), R Foundation for Statistical Computing, Vienna, Austria). Continuous variables were expressed as mean ± standard deviation (SD) or median (interval between quartiles, IQR). Categorical variables were expressed by numbers (%). The Shapiro‐Wilk test was used for testing normality. Between‐group differences were analyzed by independent‐samples t‐test for normally distributed continuous variables, and by the Mann‐Whitney U test for non‐normally distributed continuous variables. The Chi‐square test or Fisher's exact test was used for categorical variables. The “XGboost” algorithm was used to screen the predictors of RV myocardial strain in LVH fetuses [21]. 80% of the data were randomly selected as the training set, and the remaining 20% were used as the test set. Based on our results, 15 statistically or clinically significant echocardiographic parameters were included: LVEDD z‐score, LVEDL z‐score, RVEDD z‐score, RVEDL z‐score, RVSI, LVSI, AV z‐score, AAo z‐score, PV z‐score, MPA z‐score, RV/LV ratio, GA, type of intracardiac malformation, and presence or absence of VSD. Utilizing SHAP (SHapley Additive exPlanations) values offers consistent and locally accurate attribution values. The SHAP summary plot of the XGboost model illustrates the relationship between the high and low feature [22]. Myocardial deformation parameters of 15 randomly selected fetuses for intra‐observer and inter‐observer reproducibility were calculated using Bland‐Altman analysis and intraclass correlation coefficients (ICCs) with a 95% confidence interval. All tests were 2‐tailed, and *p* < 0.05 was considered statistically significant.

## 3 Results

### 3.1 Clinical Characteristicsin Study Population

A total of 162 fetuses were enrolled in this study, including 81 LVH fetuses and 81 controls. The baseline clinical characteristics are summarized in Table 1. Cardiac anomalies identified in LVH fetuses included hypoplastic left heart syndrome (HLHS) (48/81, 59.2%), unbalanced atrioventricular septal defect (right ventricular dominant) (18/81 cases, 22.2%), and double outlet right ventricle (DORV) (15/81, 18.5%). Fetal ventricular septal defects were present in 25.9% (21/81) of LVH cases. There were statistically significant differences in the pregnancy histories, including gravidity and parity between the LVH group and the normal control group. However, there were no statistically significant differences in the age, BMI, and gestational age at examination between the two groups (Table 1).

**Table 1:** Clinical characteristics in study population.

Variable	Control group	LVH group	*p*
	(*n* = 81)	(*n* = 81)	
**Maternal characteristic**			
Age (years)	30.83 ± 4.12	29.16 ± 5.33	0.090
BMI (kg/m^2^)	24.69 ± 3.40	23.26 ± 5.14	0.080
Gestational Age(weeks)	24.6 (23.5–26.5)	24.6 (23.4–27.1)	0.980
Obstetric history			
Gravidity			0.020
1	31 (38.3%)	24 (29.6%)	
2	29 (35.8%)	21 (25.9%)	
≥3	21 (26.0%)	36 (44.5%)	
Parity			0.010
0	52 (64.2%)	35 (43.2%)	
1	29 (35.8%)	41 (50.6%)	
2	0 (0.0%)	5 (6.2%)	
Abortion			0.070
0	47 (58.0%)	39 (48.1%)	
1	23 (28.4%)	31 (38.3%)	
2	8 (9.9%)	5 (6.2%)	
≥3	3 (3.7%)	6 (7.4%)	
**LVH with VSD**		21 (25.9%)	
**Intracardiac malformations**			
DORV		18 (22.2%)	
R‐uAVSD		15 (18.5%)	
HLHS		48 (59.2%)	

*Note:* Data are presented as mean ± SD or *n* (%).

Abbreviations: BMI, body mass index; DORV, double outlet right ventricle; HLHS, hypoplastic left heart syndrome; R‐uAVSD, right‐dominant unbalanced atrioventricular septal defect; VSD, ventricular septal defect.

### 3.2 Conventional Echocardiographic Characteristics for RV in LVH Fetuses

In LVH fetuses, the z‐scores for LVEDD, LVEDL, AV, and AAo were significantly lower than those in the control group (*p* < 0.001). In comparison with the control group, the RVEDL z‐score and RVSI decreased (*p* < 0.001) and the z‐score for RVEDD increased, indicating that the RV cavity was more spherical. The z‐scores for the LPA and RPA decreased, whereas that for the DA increased (*p* < 0.001). No significant differences were observed in the peak systolic velocities of the AV, PV, and DA between the LVH and control groups (Table 2).

**Table 2:** Conventional echocardiographic characteristics of the study population.

Variable	Control group	LVH group	*p*
	(*n* = 81)	(*n* = 81)	
**Morphological parameters**			
RVSI	1.73 ± 0.30	1.44 ± 0.30	**<0.001**
LVSI	2.01 ± 0.24	2.05 ± 0.60	0.868
RV/LV ratio	1.02 ± 1.19	2.40 ± 1.19	**<0.001**
LVEDD z‐score	0.28 (−0.10, 0.70)	−4.06 (‐5.27, −2.31)	**<0.001**
LVEDL z‐score	0.1 (−0.40, 0.55)	−4.57 (−6.01, −3.08)	**<0.001**
RVEDD z‐score	0.01 (−0.30, 0.45)	1.02 (−0.12, 1.78)	**<0.001**
RVEDL z‐score	−0.11 (−0.60, 0.23)	−0.75 (−1.55, −0.22)	**<0.001**
FO (cm)	0.40 (0.30, 0.51)	0.50 (0.44, 0.56)	**<0.001**
PV z‐score	0.19 (−0.40, 0.71)	0.38 (−0.35, 1.09)	0.898
MPA z‐score	0.63 (0.20, 1.04)	0.57 (0.47, 0.65)	0.587
LPA z‐score	0.26 (−0.10, 0.54)	−0.53 (−1.19, −0.03)	**<0.001**
RPA z‐score	0.12 (−0.30, 0.52)	−0.78 (−1.47, −0.06)	**<0.001**
DA z‐score	−0.07 (−0.50 to 0.25)	0.59 (−0.47, 0.93)	**0.001**
AAo z‐score	0.15 (−0.10, 0.46)	−2.84 (−4.3, −1.83)	**<0.001**
AV z‐score	0.16 (−0.10, 0.6)	−2.62 (−4.34, −1.52)	**<0.001**
**Flow characteristics**			
PV PSV (cm/s)	71.71 ± 12.57	78.1 ± 16.92	0.168
AV PSV (cm/s)	95.39 ± 16.69	97.27 ± 24.59	0.468
DA PSV (cm/s)	95.16 ± 8.53	109.37 ± 53.22	0.863
FHR (bmp)	148.50 ± 7.43	148.65 ± 9.88	0.567

*Note:* Data are presented as interquartile range.

Abbreviations: AAo, ascending aorta diameter; AV, aortic valve diameter; DA, ductus arteriosus; FHR, fetal heart rate; LPA, pulmonary artery diameter; LVEDD, left ventricular end‐diastole dimension; LVEDL, left ventricular end‐diastole length; LVSI, left ventricular sphericity index; MPA, main pulmonary artery diameter; PV, pulmonary valve diameter; PSV, peak systolic velocity; RPA, pulmonary artery diameter; RVEDD, right ventricular end‐diastole dimension; RVEDL, right ventricular end‐diastole length; RVSI, right ventricular sphericity index.

### 3.3 Myocardial Deformation of RV in Fetuses With LVH

RVGLS was significantly lower in LVH fetuses than in control group fetuses (*p* < 0.001). For the RV lateral and septum walls, basal and mid‐segmental longitudinal strains were reduced in LVH fetuses compared with normal fetuses (*p* < 0.05). However, there were no significant differences in the apical segments between the two groups. Additionally, in normal fetuses, the RV lateral and septal wall longitudinal strains decreased from basal to apical (*p* < 0.05), whereas LVH fetuses exhibited similar RV strain values among the different segments without significant gradient differences (*p* > 0.05) (Table 3).

**Table 3:** Strain indices of the study population by 2D‐STE.

	Control	LVH	*p*
	(*n* = 81)	(*n* = 81)	
Global strain			
RVGLS (%)	−22.04 ± 1.81	−17.62 ± 4.47	**<0.001**
Segmental strain			
RV lateral			
Basal	−26.39 (−33.37, −19.45)*,$	−17.64 (‐25.03, −11.83)	**0.001**
Mid	−21.53 ± 13.15*,#	−16.07 ± 8.16	**0.040**
Apical	−16.62 ± 10.36 #,$	−15.37 ± 10.24	0.577
Septum			
Basal	−24.09 ± 8.86$	−16.51 ± 8.27	**<0.001**
Mid	−23.81 (−30.67, −20.55) #	−17.34 (‐21.04, −10.11)	**0.002**
Apical	−19.24 ± 5.83$,#	−18.72 ± 8.18	0.520

*Note:* Data are presented as mean ± SD, median (interquartile range). LVH, left ventricular hypoplasia; RVGLS, right ventricular global longitudinal strain;

* *p* < 0.05 basal segmental strain compared with mid segment;

^#^ *p* < 0.05 mid segmental strain compared with apical segment;

^$^ *p* < 0.05 basal segmental strain compared with apical segment.

The intraobserver and interobserver reproducibility values are summarized in Table 4. The RV segmental and global longitudinal strains displayed excellent reproducibility as reflected by their high intraclass correlation coefficients. The Bland‐Altman analysis demonstrated good intraobserver and interobserver agreement, with a small bias and narrow limits of agreement.

**Table 4:** Inter‐observer and intra‐observer reproducibility for the parameters of myocardial deformation.

	ICC (95% CI)	Bias	95%LOA
Intra‐observer (*n* = 30)			
RVGLS	0.85 (0.80–0.91)	0.04	−2.99–2.44
RV lateral			
Basal	0.88 (0.79–0.92)	0.02	−0.31–0.35
Mid	0.90 (0.83–0.97)	−0.01	−0.39–0.31
Apical	0.81 (0.76–0.89)	−0.02	−0.45–0.43
Septum			
Basal	0.80 (0.75–0.86)	0.10	−1.25–1.45
Mid	0.92 (0.81–0.96)	0.05	−0.59–0.69
Apical	0.91 (0.82–0.95)	0.04	−0.33–0.38
Inter‐observer (*n* = 30)			
RVGLS	0.88 (0.80–0.93)	0.07	−2.50–2.66
RV lateral			
Basal	0.91 (0.86–0.95)	0.02	−0.35–0.37
Mid	0.95 (0.93–0.97)	−0.08	−0.55–0.47
Apical	0.82 (0.76–0.89)	−0.02	−0.84–0.76
Septum			
Basal	0.90 (0.84–0.94)	0.02	−1.11–1.08
Mid	0.89 (0.81–0.93)	0.06	−0.59–0.71
Apical	0.88 (0.82–0.93)	0.09	−0.66–0.72

Abbreviations: ICC, intra‐class correlation coefficients; LOA, limit of agreement; RVGLS, right ventricular global longitudinal strai.

### 3.4 Predictors of RV Myocardial Strain in LVH Fetuses

#### 3.4.1 Variable Selection

**Figure 1:**
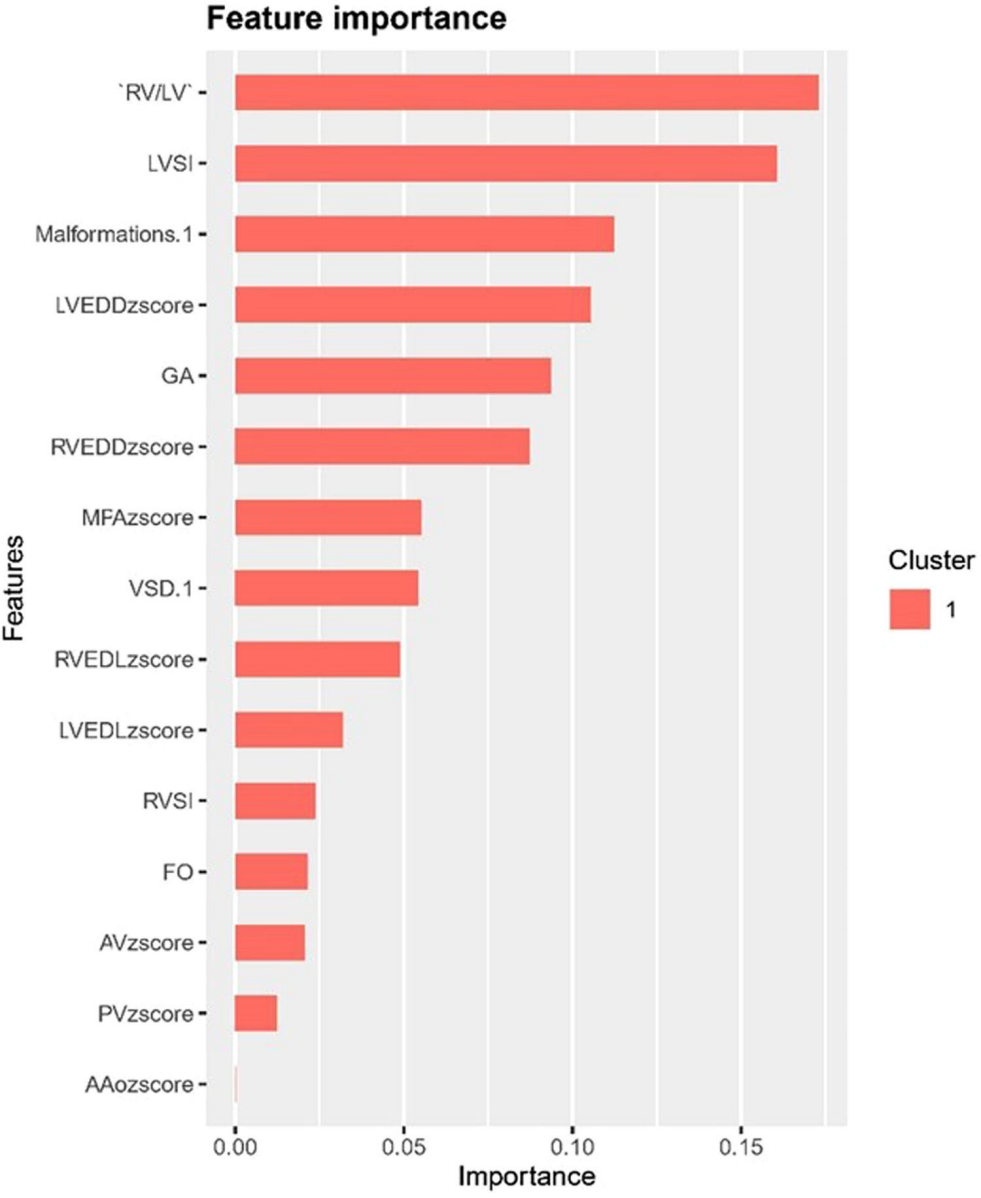
Importance matrix plot of the XGboost model. This importance matrix plot depicts the importance of each variate in the predictive model. X‐axis represents the importance score of the corresponding variable, and the Y‐axis represents the variable. The higher the score, the more susceptible RVGLS is to this variable. AV, aortic valve; DA, ductus arteriosus; GA, gestational age; LVEDD, left ventricular end‐diastole dimension; LVEDL, left ventricular end‐diastole length; LVSI, left ventricular sphericity index; Malformations.1, LVH with Double Outlet Right Ventricle; MPA, main pulmonary artery diameter; PV, pulmonary valve diameter; RVEDD, right ventricular end‐diastole dimension; RVEDL, right ventricular end‐diastole length; RVSI, right ventricular sphericity index; VSD.1, LVH with VSD.

The ranking of variables importance in “XGboost” model showed that RV/LV ratio, LVSI, combined DORV, and LVEDD z‐score had the greatest effect on RVGLS, while LVH with VSD had no significant effect on RVGLS (Figure 1).

#### 3.4.2 Model Explanation

**Figure 2:**
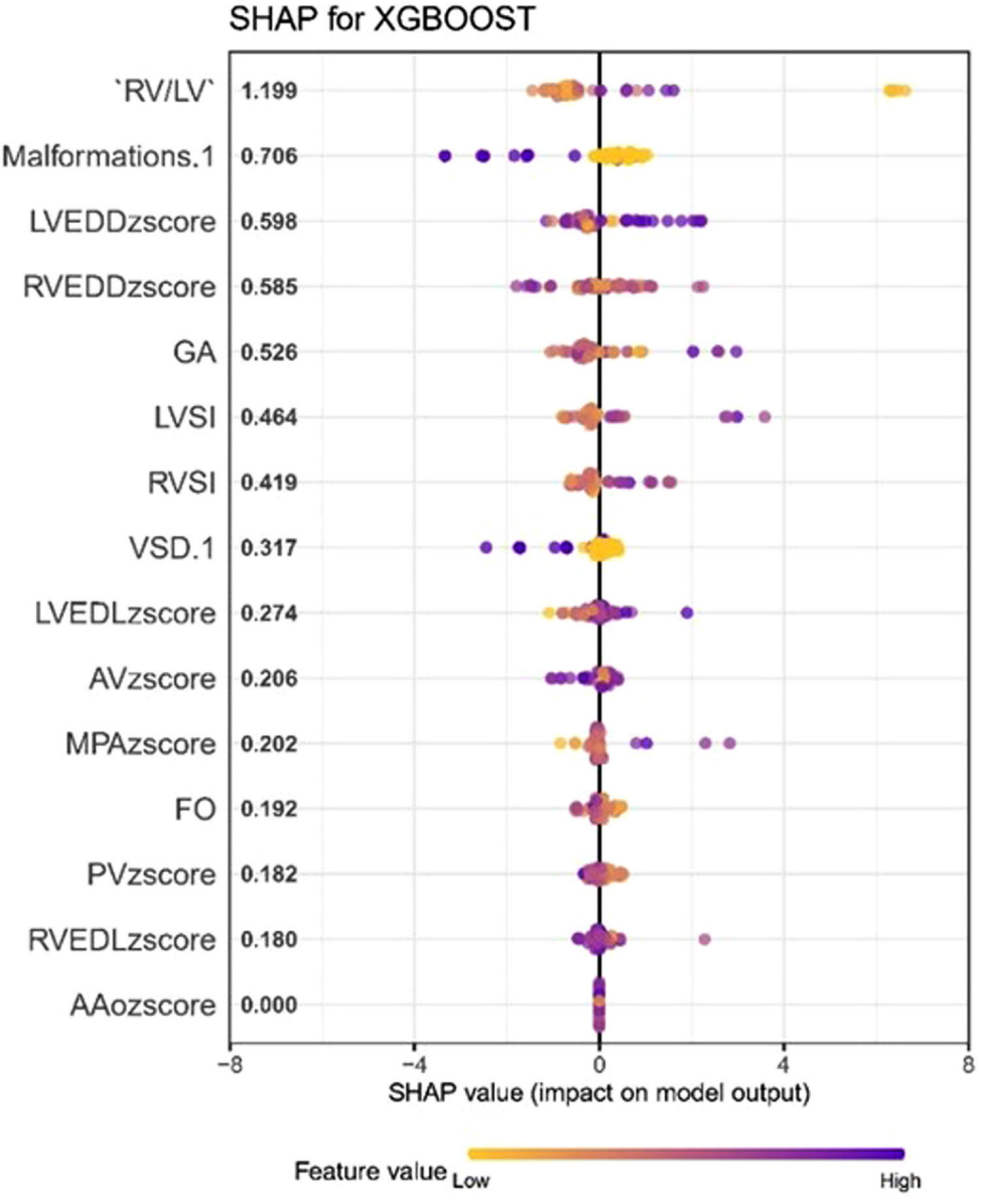
SHAP summary plot of XGboost model. The higher the SHAP value of a feature, the higher the higher the SHAP value of a feature, the greater its impact on RVGLS. A dot is created for each feature attribution value for the model of each fetus, and thus one fetus is allocated one dot on the line for each feature. Dots are colored according to the values of features for the respective fetus and accumulate vertically to depict density. Purple represents higher feature values, and yellow represents lower feature values. AV, aortic valve; DA, ductus arteriosus; GA, gestational age; LVEDD, left ventricular end‐diastole dimension; LVEDL, left ventricular end‐diastole length; LVSI, left ventricular sphericity index; Malformations.1, LVH with Double Outlet Right Ventricle; MPA, main pulmonary artery diameter; PV, pulmonary valve diameter; RVEDD, right ventricular end‐diastole dimension; RVEDL, right ventricular end‐diastole length; RVSI, right ventricular sphericity index; VSD.1, LVH with VSD.

According to the predictive model, the higher the SHAP value of a feature, the greater its impact on the RVGLS. In the SHAP plot, the impact on the RVGLS decreased in the following order: RV/LV diameter ratio, combined DORV, and LVEDD z‐scores (Figure 2).

**Figure 3:**
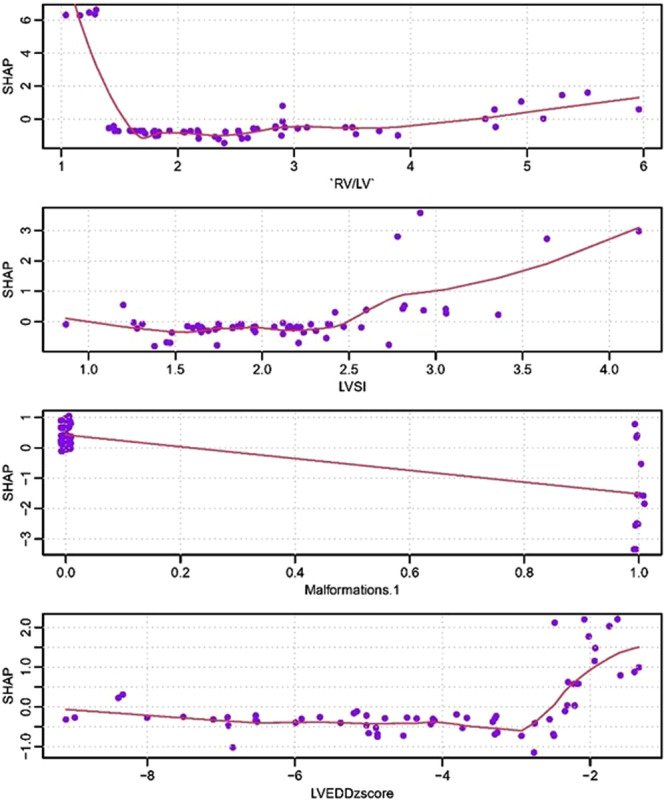
SHAP dependence plot of the XGboost model. The SHAP dependence plot shows how a single feature affects the output of the XGboost prediction model. SHAP values for specific features exceed zero, representing positively affected RVGLS values, while the SHAP value is less than zero indicates that the RVGLS value is negatively affected.

The SHAP dependency graph (Figure 3) can be employed to understand the influence of individual features on the output of the XGboost model. The y‐axis represents the SHAP values of each feature and the x‐axis denotes the variable values. Analysis of the SHAP dependency graph revealed that when LVH fetuses were combined with DORV, the absolute value of the RVGLS increased. The impact of fetal LVEDD z‐score on RVGLS values undergoes a transition from negative to positive: when LVEDD z‐score is <−2, the absolute value of RVGLS increases with the decrease of LVEDD z‐score, but when the LVEDD z‐score approaches −2, the absolute value of RVGLS decreases with the increase of LVEDD z‐score.

Furthermore, the influence of the RV/LV diameter ratio on the RVGLS exhibited a transition from a positive to negative impact. When the RV/LV ratio was close to 1, the absolute value of RVGLS decreased with an increase in RV/LV; when the RV/LV ratio was <1, the negative value of RVGLS increased with an increase in RV/LV. This comprehensive analysis, guided by SHAP values, provides insights into the intricate relationships between variables and their impact on the RVGLS within the XGboost model.

### 3.5 Bioinformatic Analysis for RV Differential Proteins in LVH Fetuses

**Figure 4:**
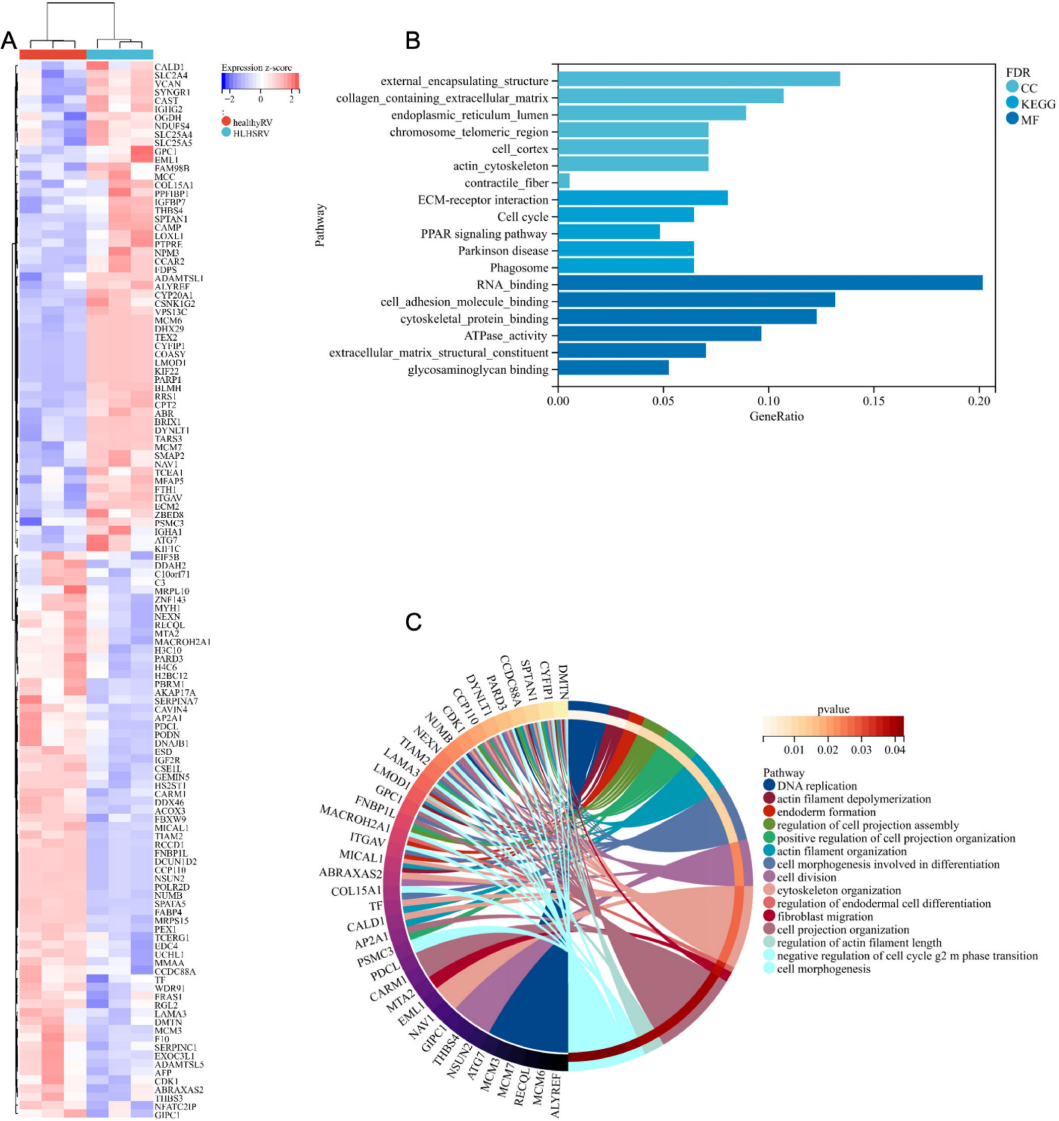
Gene ontology (GO) term and pathway enrichment analysis. (A) A comparison of right ventricular (RV) myocardial proteins in left ventricular hypoplasia (LVH) fetuses and normal samples. (B), (C). GO analysis for differential proteins from LVH and control hearts.

**Figure 5:**
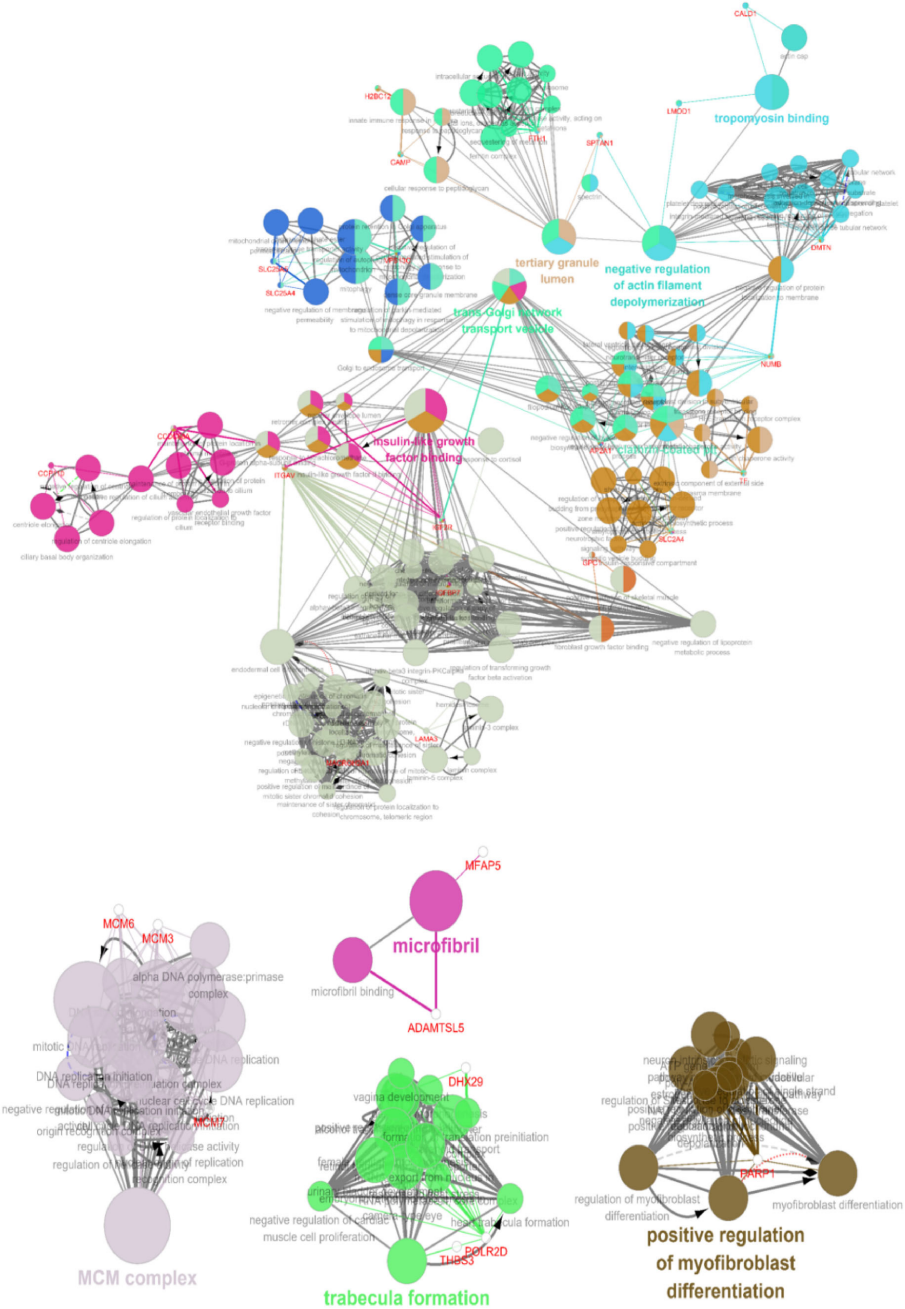
Gene ontology analysis incorporating cellular components and biological processes and KEGG significantly altered based on differentially proteins (ClueGO).

A total of 144 RV differential proteins in three LVH fetuses were screened out, in which 68 proteins were upregulated, 76 were downregulated compared with normal fetal specimens. The detailed GO analysis was summarized in Table S1 Supp Info. GO analysis showed that these differential proteins mainly constituted the structures of cell outer capsule, collagen extracellular matrix, endoplasmic reticulum lumen, chromosomal telomeres, contractile fiber, cytoskeleton, DNA replication licensing factor complex protein (MCM), etc, participated in actin fiber depolymerization, endoderm formation, cell division, regulation of cell cycle G2‐M transition and other biological processes; related to RNA binding, cell adhesion molecule binding, cytoskeletal protein and other molecular functions. KEGG analysis showed that differential proteins were involved in extracellular matrix receptor interaction, cell cycle and other signaling pathways (Figure 4). The ClueGO plugin for Cytoscape was utilized for centralized analysis and evaluation of biological networks, including endodermal cell differentiation, negative regulation of actin filament depolymerization, tropomyosin binding, trabecula formation, MCM complex, among others (Bonferroni corrected *p* value < 0.05; Figure 5). The ClueGO analysis represents these biological networks as interconnected nodes, with each node corresponding to a gene, protein, or pathway. The clusters identified in the analysis reflect related biological processes, and the edges connecting the nodes illustrate the interactions between these processes. The significant clusters or modules revealed by the analysis suggest potential molecular mechanisms involved in cardiac remodeling and RV adaptation in fetuses with LVH. Significant clusters or modules that are identified in the analysis suggest potential molecular mechanisms involved in cardiac remodeling and right ventricular adaptation in LVH fetuses. The clustering of processes like actin filament regulation, tropomyosin binding, and MCM complex would point to a strong emphasis on maintaining cardiomyocyte integrity, cytoskeletal function, and cell cycle regulation during cardiac development.

## 4 Discussion

In this study, conventional fetal echocardiography and STI were used to comprehensively evaluate RV morphology and cardiac function. Additionally, proteomics analysis was performed to elucidate RV cardiomyocytes proteins in LVH fetuses. The main findings of this study were as follows: 1) compared with controls, RV morphology was more spherical and RVGLS were reduced in LVH fetuses; 2) there appears to be a threshold effect where the degree of LV dilation initially worsens RV strain, but beyond a certain point, the relationship between LVEDD and RV strain may diminish. 3) Our proteomics analysis revealed that RV function adaptation in LVH fetuses involves alterations in cell cycle‐related proteins and cytoskeletal proteins, suggesting phenotypic changes in RV cardiomyocytes similar to those observed in LVH‐associated left heart dysfunction.

### 4.1 Changes in Cardiac Geometry and Long‐Axis Contractility in Fetal LVH

In LVH fetuses, conventional echocardiography indicated that compared to normal fetuses, RV cavity was closer to spherical in LVH fetuses, RVEDD z‐score and RV/LV ratio increased, RVSI decreased. The change in the geometry of right heart cavity reflects adaptation to chronic volume loading, which may normalize the preload through eccentric remodeling. Previous experimental studies have shown that long‐term RV overload causes ventricular remodeling and dysfuntion [23, 24].

The RVGLS was reduced, suggesting long‐axis RV dysfunction in fetuses with LVH. First, ventricular global longitudinal strain also was affected by chronic volume loading [25].

Second, RV function adaption is associated with biventricular interactions. The anatomy of the ventricular‐ventricular interaction is based on the common pericardium, interventricular septum, and certain myocardial fibers that extend between the ventricles, such as the RV outflow tract connecting to the LV free wall myocardium [26, 27, 28, 29, 30]. The normal RV ejection is intricately linked to the LV. During systolic phase, LV assist RV contraction by stretching the RV free wall through the interventricular septum [28, 31, 32]. In the experimental model, LV contraction generates 20% to 40% for right cardiac output [33, 34]. Consequently, it can be reasonably inferred that LVH fetuses with abnormal LV development are likely to experience significant RV dysfunction. By combining XGBoost with SHAP values, we found that the LVEDD z‐score, RV/LV diameter ratio and LVSI exhibited a more complex relationship with RVGLS. This suggests that there is a threshold effect in which the extent of LV dilation may initially exacerbate RV strain but, beyond a certain point, the relationship between LVEDD and RV strain may become less pronounced. This result was consistent with previous study that Doppler technology was used to evaluate the right heart function of HLHS fetuses [35]. Based on the previous discoveries and the outcome of this investigation, it can be deduced that the LV morphology played a crucial role in influencing the RV function and the prognosis of fetuses with LVH. Interestingly, the presence of LVH with VSD did not significantly affect RVGLS in this model. This finding suggests that while VSD is an important structural abnormality, it may not independently influence the RVGLS in fetuses with LVH.

Normal RV mainly consists of circumferential and longitudinal fibers; unlike the LV, there is no obvious three‐layer myocardium, and the longitudinal fibers were aligned base to the apex. This structural characteristic enables RV to shorten longitudinally [27]. However, both RV basal and middle segment longitudinal strain decreased, and RV segmental strain of did not show a trend of basal‐apex gradient in LVH fetuses. So, the third reason for impaired RV function in LVH fetuses may the arrangement of RV myocardial fibers changed in this pathological state.

### 4.2 RV Differential Proteins in LVH Fetuses

To further explain mechanism of RV function adaption, we utilized proteomics to acquire insight into the molecular basis for RV function and structural changes. Specifically, we observed alterations in the expression of MCM complexes, which necessary for DNA replication in eukaryotic cells [36]. Past research predominantly concentrated on LV cardiomyocytes in LVH fetuses. Earlier investigation using single‐cell RNA sequencing of induced pluripotent stem cells in three LVH patients and three controls demonstrated that the LV cardiomyocytes in HLHS patients prematurely exited the cell cycle, preventing the response of growth and development signals, resulting in accumulation of DNA damage and increased cell apoptosis [37]. These observations suggest cell cycle abnormalities are not limited to the left heart but also extend to the RV cardiomyocytes.

Another important finding was the alteration of the expression of various cytoskeletal proteins in RV tissues, including NEXN, CALD1, MFAP5, LMOD1. These proteins play an important role in various parts of myocardial contraction, NEXN involved in the maintenance of Z line and sarcomere integrity [38]. CALD is essential for cardiac morphogenesis and function, loss of CALD expression could lead to ventricular dysfunction [39]. The upregulated expression of MFAP5 was found to be in a mouse model of cardiac remodeling [40]. These results indicated that the programs of RV cardiomyocytes sarcomere and cardiac contraction display phenotypic changes in LVH fetuses.

The results of this study suggested abnormal expression of extracellular matrix proteins in the RV tissues, including IGTVA, VCAN, GPC1, THBS3/4, and COL15A1. We discovered that the expression of THBS3 in LVH fetuses decreased, but THBS4 increased. It is worthy mentioning that THBS protein is selectively expressed during embryonic development, and studies demonstrated that THBS3 could deteriorate muscle membrane instability by decreasing integrin function and THBS4 expression augmented during ventricular overload, myocardial hypertrophy, and heart failure [41, 42]. In addition, prior research suggests that ventricular overload derived upregulation of COL15A1 in cardiomyocytes and endothelial cells, which was consistent with our study [43]. These findings provide a molecular explanation for cardiac remodeling and reduced cardiac function in LVH fetuses.

Furthermore, ClueGO plugin for Cytoscape was utilized for centralized analysis and evaluation of biological networks. Our result found that the clustering of processes such as actin filament regulation, tropomyosin binding, and MCM complex formation suggests a crucial focus on maintaining cardiomyocyte integrity, supporting cytoskeletal function, and regulating the cell cycle during cardiac development. These processes are integral to ensuring proper contractility and structural stability of cardiomyocytes, which are essential for normal heart function. Actin filaments and tropomyosin play a critical role in the sarcomeric structure, facilitating muscle contraction, while the MCM complex is pivotal for DNA replication and cell cycle progression, particularly in the proliferating cardiomyocytes during early stages of development. Collectively, these pathways highlight the importance of coordinated molecular events that not only preserve the structural framework of cardiomyocytes but also regulate their division and differentiation, ensuring proper heart formation and function.

Despite major improvements that occurred over the last 2 decades in prenatal diagnosis and surgical management, fetuses with LVH that carry substantial morbidity and mortality. RV function is an important factor of successfully navigating the single ventricle palliative pathway, although identifying subtle changes in function over time can be a challenge. Our findings shed light on a novel perspective that myogenic developmental abnormalities are not limited to the left heart but also extend to the RV cardiomyocytes during the embryonic stage, which may contribute to enhanced risk stratification for RV systolic dysfunction, and potentially provide significant advice on prenatal counseling, prognostication, and management of fetuses in LVH.

### 4.3 Strengths and Limitations

The primary strength of our study is the identification of an association between subtle changes in RV function and myogenic developmental abnormalities. In our study, impaired RV myocardial function was observed in fetuses with LVH using 2D‐STI, which can quantify cardiac structural and functional changes at an early stage. In addition, the LV dimension is a crucial determinant of RV function, which reinforces RV–LV interactions and the potential influence of prenatal RV function on the prognosis and outcomes in fetuses diagnosed with LVH. Finally, our proteomic analysis provided information regarding the intrinsic myocardial structure, demonstrating that this possible additional explanation reduced the RV performance.

Our study had several limitations. Although a deeper understanding of the mechanistic underpinnings of LVH has been achieved, the sample size is relatively small. In addition, our data cannot be used for survival analysis because most families chose to terminate pregnancy when the fetuses were diagnosed with LVH during the second‐trimester screening.

## 5 Conclusion

According to our findings, RV function is impaired in fetuses with LVH. We also identified the LV dimension as a crucial determinant of RV function. To clarify the underlying pathological mechanism, we sequenced protein profiles and identified proteins linked to key cellular functions, such as the cell cycle, cytoskeleton, and extracellular matrix, which provided valuable insights into ventricular remodeling and its impact on cardiac function.

## Author Contributions

Conceptualization: Li Zhang and Mingxing Xie, Writing – original draft preparation: Jing Ma and Sushan Xiao, Writing – review and editing, Jing Ma, Sushan Xiao and Liu Hong, Methodology: Jing Ma, Sushan Xiao and Yi Zhang, Software: Juanjuan Liu, Jiawei Shi and Li Cui, Investigation: Jing Ma and Sushan Xiao, Supervision: Haiyan Cao Li Zhang, Mingxing Xie, Funding acquisition, Li Zhang, Mingxing Xie, Haiyan Cao. All authors have read and agreed to the published version of the manuscript.

## Conflicts of Interest

The authors declare no conflicts of interest.

## Transparency Statement

The lead author Li Zhang affirms that this manuscript is an honest, accurate, and transparent account of the study being reported; that no important aspects of the study have been omitted; and that any discrepancies from the study as planned (and, if relevant, registered) have been explained.

## Supporting information

Table S1SuppInfo provides detailed functional annotations for 128 of these proteins. The analysis categorizes proteins according to their involvement in biological processes, molecular functions, and cellular components. Each entry includes the protein name, its unique identifier, the specific GO category (e.g., GO Process, GO Function, GO Component), the corresponding GO term ID, and a detailed term description. These data provide in‐depth insights into the molecular pathways and biological functions that may be altered in the right ventricle of LVH fetuses.

## Data Availability

The authors confirm that the data supporting the findings of this study are available within the article and its supporting materials.
